# Nanotechnology in Chronic Pain Relief

**DOI:** 10.3389/fbioe.2020.00682

**Published:** 2020-06-19

**Authors:** Jing Chen, Teng Jin, Hua Zhang

**Affiliations:** ^1^School of Life Sciences, Shanghai University, Shanghai, China; ^2^Department of Radiology, Union Hospital, Tongji Medical College, Huazhong University of Science and Technology, Wuhan, China; ^3^Department of Radiology, The Affiliated Hospital of Qingdao University, Qingdao, China

**Keywords:** nanotechnology, chronic pain, inflammation, drugs, targeting

## Abstract

Increasing awareness of chronic pain due to both injury and disease have encouraged drug companies and pharmaceutical researchers alike to design and fabricate better, more specific drugs for pain relief. However, overuse of clinically available pain medication has caused a multitude of negative repercussions, including drug tolerance, addiction, and other severe side effects, which can prolong suffering and reduce pain mediation. Applications of nanotechnology to the field of drug delivery has sought to enhance the treatment efficiency, lower side effects, and mitigate the formation of tolerance. The use of nanomaterials has several advantages for chronic pain relief, such as controlled release, prolonged circulation time, and limited side effects. With the development of nanotechnology, strategies for chronic pain relief have also bourgeoned utilizing a variety of nanomaterials and targeting surface modifications. In addition to using these materials as carriers for drug delivery, nanomaterials can also be designed to have inherent properties that relieve chronic pain. This minireview covers the current status of designed nanomaterials for pain relief and provides a discussion of future considerations for nanotechnology designed for relieving chronic pain.

## Introduction

Chronic pain is characterized by enhanced responses to different external stimuli, also known as hyperalgesia, and is induced by inflammation following injury (Ji et al., 2014). When damage or inflammation occurs, mediators such as prostaglandins, cytokines, chemokines, neuropeptides, and nerve growth factor (NGF), are released (Zhang and An, 2007). These mediators maintain pain signaling that starts in the periphery and results in both peripheral and central sensitization, ultimately contributing to chronic pain. From the tremendous efforts of researchers in the fields of neurology and signaling, we have a deeper understanding of the mechanisms that drive pain. Additionally, chronic pain from a variety of sources, including injuries and disease, has promoted the development of targeted therapies (Mantyh et al., 2002; Binder, 2007; Said, 2007; Francis et al., 2008).

Current methods of pain relief and enhanced quality of life predominantly rely on surgery (Ducic et al., 2008), medication (Volkow et al., 2018), physical therapy (Ambrose and Golightly, 2015), and psychological therapy (De Williams et al., 2012). The use of medications, including opioid drugs (Sullivan and Howe, 2013; Ballantyne and Sullivan, 2015) and non-opioid drugs (Kaye et al., 2018), has increased significantly over the last several decades. Extensive use of medication has been associated with severe side effects, including drug addiction (Pohl and Smith, 2012), tolerance (Zhuo, 2016), abuse (Vowles et al., 2015; Volkow and McLellan, 2016), and even death. These significant drawbacks of clinically available drugs have shifted the focus of drug development on improving the targeting of drugs, reducing side effects, and prolonging the release of the active compounds (Gao and Ji, 2010). However, due to their rapid metabolism, these current formulations are challenging to manufacture reproducibly, and the required dosing can cause poorly tolerated physical side effects.

**FIGURE 1:**
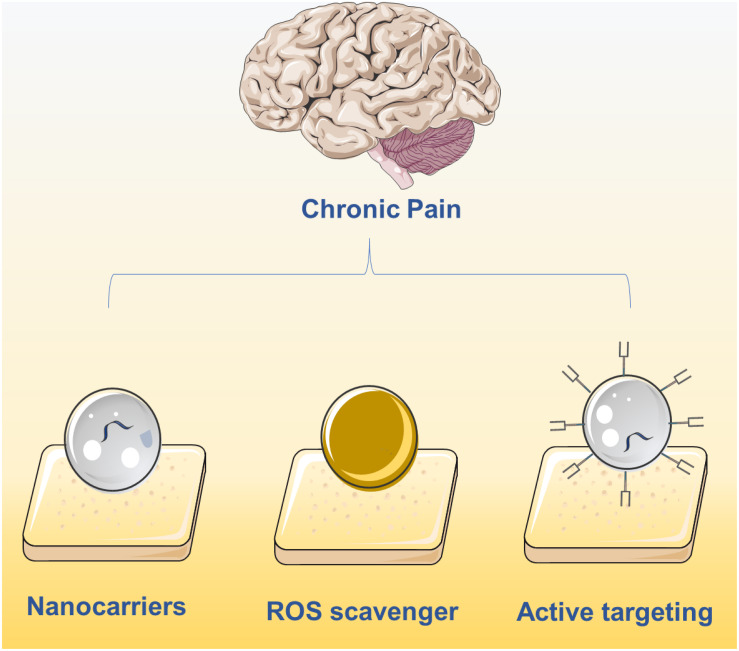
Current main strategies on chronic pain relief using nanotechnology including the delivery of drugs using nanocarriers, active targeting nanocarriers and ROS clearance via nanomaterials.

The integration of pharmacological sciences with nanotechnology has been a key step toward creating more effective drugs for chronic pain with fewer negative implications (Feynman, 1960). With the advent of nanotechnology, the field of drug delivery has undergone extensive development resulting in several nanomaterials being approved for clinical use (Ventola, 2017). Compared with traditional formulations, nanomaterials can be efficiently loaded with drugs (Farokhzad and Langer, 2009), protect the stability of protein-based drugs (Xu et al., 2019), sustain controlled release with prolonged circulation time (Blanco et al., 2015), and are also designed to be highly biocompatible (Nyström and Fadeel, 2012). In the field of chronic pain relief, nanomaterials have been developed explicitly for the targeted delivery and release of pain medication. Inspired by the first Food and Drug Administration (FDA)-approved nanodrug Doxil (Barenholz, 2012), nanotechnology is being applied to many biomedical applications, but with a limited focus on chronic pain relief. In this minireview, we cover the development of medications for chronic pain relief that employ nanotechnology, including targeted and non-targeted nanomaterials, and provide perspective for future applications of nanotechnology in pain relief (Figure 1).

## Non-Targeted Nanomaterials for Pain Relief

Nanomaterials can be designated as organic, inorganic, and metal-organic nanomaterials based on their components. All three categories of nanomaterials have been used as controlled release delivery systems to minimize side effects and promote treatment efficacy for pain medication. Nanomaterials can be used to encapsulate both free molecules and protein-based drugs to increase blood circulation time with sustained, controlled release, resulting in long-lasting pain relief with minimal side effects. In this section, we will present the development of organic and inorganic non-targeted nanomaterials, which have been broadly applied to several pain relief drugs.

When introducing nanomaterials into a clinical application, a major preliminary concern is the biocompatibility of the proposed nanomaterial. Consequently, nanomaterials that have already been approved by the FDA are generally the first to be considered by researchers. FDA approved nanomaterials are mainly organic in nature such as liposomes (Koudelka and Turánek, 2012), PLGA (Makadia and Siegel, 2011), and other carbon based polymer nanomaterials (Palazzolo et al., 2018). Several inorganic nanomaterials have also been approved, but remain in the minority (Urie et al., 2018).

Liposomes are especially attractive since they are derived from cellular-like lipids, making them extremely biocompatible, and are relatively well studied. A number of liposome formulations have been the focus of emerging clinical trials. For example, PEGylated-liposomes have been used to encapsulate and enhance the accumulation of zoledronic acid (ZOL), an inhibitor of the ras-dependent Erk-mediated pathway, for the treatment of neuropathic pain (Caraglia et al., 2013). Caraglia et al. proved that this liposomal based delivery system passed across the blood brain barrier (BBB), promoting its ability to release ZOL for efficient pain mitigation. Likewise, Smith et al. (2006) encapsulated the drug hydromorphone using the liposomes and tested its neuropathic pain relief ability in rats. As expected, liposomal delivery enabled prolonged pain relief with only a single injection.

Although liposomes have many advantages and benefit from established synthesis procedures, the next generation of nanomaterials is focused on a myriad of tunable features including size, surface properties, responsiveness, controlled circulation time, high loading efficiency, and the ability to target specific tissues. Durán-Lobato et al. (2015) compared PEG-modified lipid nanomaterials to chitosan-modified lipid nanomaterials and PLGA nanomaterials for their ability to deliver cannabinoids via oral administration. The three biocompatible nanomaterials varied in their performance, providing design criteria for the development of nanomaterials for pain relief drug delivery.

The overproduction of reactive oxygen species (ROS) at sites of inflammation can result in chronic pain; consequently, nanomaterials that consume ROS are a promising avenue for pain relief (Gwak et al., 2013). Along these lines, Liu et al. (2013) employed fullerol nanomaterials, which are known to consume ROS, to protect inflammatory sites and relieve the pain.

Integrating an FDA-approved nanomaterial PLGA with bupivacaine, Garcia et al. (2011) studied its drug release mechanism and ability to relieve pain. Likewise, Shen et al. (2013) synthesized a new combination of nanomaterial, naocurcumin, composed of PLGA and curcumin to attenuate morphine tolerance. Naocurcumin was orally administrated to mice revealing excellent biocompatibility and ability to mitigate tolerance to morphine. Apart from small molecule drug delivery, biomedicine and nanotechnology have also been focused on the delivery of biomolecules such as Ribonucleic Acid (RNA). Based on the ability of p38 small interfering RNA (p38 siRNA) to assuage neuropathic pain, Shin et al. (2018) used PLGA nanomaterials to encapsulate the p38 siRNA enhancing its stability and slow release for pain alleviation.

In addition to organic nanomaterials, inorganic nanomaterials have also been designed and applied to address chronic pain. Compared with their organic counterparts, inorganic nanomaterials are generally more stable, ensuring prolonged circulation, and can be designed to have more physicochemical properties, such as controlled drug release via stimulation by an external field. Recently, Song et al. (2015) published on a graphene oxide (GO) based nano-system for neuropathic pain relief. Specifically, these GO nanomaterials provided a large surface area to load lidocaine and thalidomide, enhancing the capacity of these drugs to relieve neuropathic pain. In a separate study of inorganic nanomaterials, Wu et al. (2017) demonstrated that ultra-small magnetic iron oxide particles exhibited dose-dependent analgesic effects for the treatment of chronic pain. Using hydroxyapatite nanomaterials, which are biocompatible and have been approved for use in bone-related diseases, Gu et al. (2012) encapsulated and efficiently delivered NR2B-siRNA into mice via intrathecal injection. These nanoparticles exhibited statistical levels of pain mitigation that motivate the future development of materials for siRNA delivery.

## Targeted Nanomaterials for Pain Relief

Despite the advantages of using nanomaterials to encapsulate drugs for the alleviation of chronic pain, their limited treatment efficacy has drawn attention to the critical need to develop more promising solutions. Since the causes of chronic pain are variable, the requirements for drug type and dosing differ depending on the treatment intervention. Enhancing the concentration of the drug at the indented site of action is one way to increase the efficacy of the treatment and minimize off target effects. Modification of nanomaterials using targeting substances such as peptides and antibodies can help achieve this site-specific targeting. Additionally, the route of administration is a critical consideration for the use of these targeted materials in their corresponding chronic pain models. For example, exposed sites of chronic pain on the skin can be topically treated using a smear or spray, whereas an injection through the dorsal root ganglion is more suitable for chronic spinal nerve pain. Internal injury or disease may benefit from oral, intranasal, intramuscular, or intravenous injections, depending on the source and location of the pain. In the following section, we will highlight research focused on targeted strategies to promote efficient chronic pain treatment.

Surface modification of nanomaterials is a simple yet effective way to enhance the location-specific absorption of delivered drugs. For example, Hoekman et al. (2014) synthesized opioid fentanyl-encapsulated liposomes with integrin targeting motifs, revealing increased stability, enhanced analgesic ability, and reduced plasma drug exposure after aerosol administration. Likewise, Tosi et al. (2007) designed polyester-based nanoparticles consisting of PLGA and glycosylated heptapeptides for the delivery of the opioid agonist loperamide. Excitingly, these nanoparticles not only crossed the BBB via peptide targeting but also exhibited sustained release of loperamide. Lalani et al. (2015) modified PLGA nanomaterials with lactoferrin and transferrin as the ligands, ensuring brain targeting for better pain relief. Additionally, Patel et al. (2012) showed enhanced tizanidine HCl delivery to the brain using surface-modified thiolated chitosan nanomaterials when they were administered intranasally.

Pain evoked by cancer, as well as some cancer medications, has drawn substantial attention from researchers studying pain relief. To address cancer-induced bone pain, Gdowski et al. (2017) modified PLGA with the bone microenvironment targeting amino-bisphosphonate, to deliver cabazitaxel for efficient pain relief in a bone metastatic prostate cancer model. To mimic the endogenous response in inflammatory sites, Hua et al. designed opioid loaded liposomes with the capacity to target inflammatory environments and release the corresponding drugs for simultaneous pain relief (Hua and Cabot, 2013). Important research described by Ramírez-García et al. (2019) reveals that their novel pH-responsive nanomaterials with the ability to target neurokinin-1 receptors (NK_1_R) in the endosome display excellent capacity for preventing chronic pain. The accumulation of these pH-responsive nanomaterials in NK_1_R-containing endosomes resulted in sustained chronic pain relief. To date, targeted nanomaterial design is mostly focused on modification with targeting ligands, but other strategies for active targeting remain largely unexplored, which will be presented in the perspective section.

## Discussion and Perspective

**FIGURE 2:**
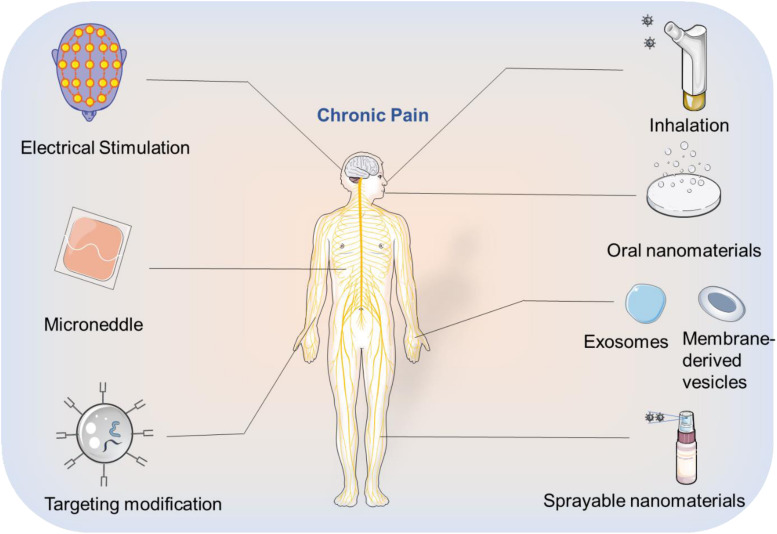
Future outlooks that can be taken up by using nanotechnology for chronic pain relief.

The urgent demand for chronic pain relief has highlighted the need for advancements in nanotechnology and pharmaceutical science, attracting attention from scientists in materials science, biomaterials, and chemistry. After decades of progress, the use of nanotechnology for chronic pain relief has been regarded as promising, prompting several clinical trials. However, the development of these smart nanomaterials is still in its infancy, with many unexplored opportunities. This minireview aims to highlight current difficulties in the pharmaceutical field and the advantages of applying nanotechnology to address these shortcomings. Herein, we have detailed some representative examples of organic and inorganic nanomaterials that facilitate controlled release, passive and active targeting, and the utilization of external energy fields. Based on these previous studies, we would like to provide some perspective thoughts on nanomaterial design for chronic pain relief (Figure 2):

(1) Oral administration of drugs poses a series of biological barriers requiring enhanced stability in the stomach, followed by the ability to be absorbed and be effective. Therefore, careful consideration of size, surface charge, and whether to modify the targeted ligands are necessary when designing a nanomaterial for this application. Moreover, non-conventional nanomaterials such as exosomes, cell membrane-derived vesicles (Van Dommelen et al., 2012) which have the targeting ability could also be investigated for oral drug delivery.
(2) Since each drug has its own kinetic profile, it is necessary to design appropriate nanomaterials to achieve a balance between drug release rate and drug action time. Additionally, combining interfering RNA with nanotechnology may prove to be an important strategy for pain mitigation.
(3) To improve therapeutic effects while minimizing side effects, a balance between the drug load and the release rate needs to be achieved. Similarly, improving the targeting efficiency of nanocarriers will reduce off-target effects by minimizing exposure to non-targeted organs during circulation.
(4) The phenomenon of protein coronas (Mahmoudi et al., 2016) has been attributed to the low efficacy of targeted nanomaterials, as serum proteins bind to the surface resulting in masking of the targeting epitopes and rapid clearance by phagocytic cells. Avoiding the formation of protein coronas is a critical avenue of research for the advancement of all nanomaterials for drug delivery. One proven method to mitigate corona formation is to modify the surface of nanomaterials with PEG.
(5) In pharmacology, the rapid development of multi-target drugs (Malek et al., 2015) has greatly improved the therapeutic outcome of chronic pain. Integrating nanotechnology with these enhanced formulations could allow for multi-drug co-delivery or their targeted release multiple sites. These methods have the capability to significantly reduce side effects, improve therapeutic outcomes, and reduce drug tolerization.
(6) Although nanomaterials have experienced rapid developmental growth over the past few decades, there is an urgency to ensure the safety of these materials and establish a unified evaluation standard.
(7) Although theranostic is a popular concept in the field of cancer, treatments for chronic pain treatment lack an intuitive imaging modality to monitor treatment progression and directly visualize therapeutic effects. Luminescent nanomaterials (Park et al., 2009) could be introduced into the field to achieve the integration of diagnosis and treatment, similar to their use in cancer therapeutics.
(8) The energy-conversion properties of inorganic nanomaterials can be employed to treat chronic pain, such as generating electrical signals when stimulated by light, (Tang et al., 2018) subsequently activating or inhibiting ion channels from playing an analgesic role. Additionally, magnetic nanomaterials (Zhang et al., 2016) can also use external magnetic fields to achieve targeted magnetic therapy.

(9) Microneedles (Kaushik et al., 2001) can be applied to the field of analgesia, where nanomaterials are integrated into the front end of the device to greatly reduce the pain caused by injecting drugs to patients.
(10) Inhalation of apposite nanomaterials that encapsulate drugs could be leveraged for chronic pain relief, especially lung-related pain.

As a collaboration between many fields of study, we believe that the application of nanotechnology to address chronic pain has a bright future. We hope that this field will develop steadily, with clinical relevance, to improve the quality of life for patients with chronic pain.

## Author Contributions

All the authors contributed to the writing of the manuscript.

## Conflict of Interest

The authors declare that the research was conducted in the absence of any commercial or financial relationships that could be construed as a potential conflict of interest.

## References

[B1] Ambrose K. R. Golightly Y. M. (2015). Physical exercise as non-pharmacological treatment of chronic pain: why and when. *Best Pract. Res. Clin. Rheumatol.* 29 120–130. 10.1016/j.berh.2015.04.022 26267006 PMC4534717

[B2] Ballantyne J. C. Sullivan M. D. (2015). Intensity of chronic pain—the wrong metric. *N. Engl. J. Med.* 373 2098–2099. 10.1056/nejmp1507136 26605926

[B3] Barenholz Y. C. (2012). Doxil^®^ —the first FDA-approved nano-drug: lessons learned. *J. Control. Release* 160 117–134. 10.1016/j.jconrel.2012.03.020 22484195

[B4] Binder A. I. (2007). Cervical spondylosis and neck pain. *BMJ* 334 527–531. 10.1136/bmj.39127.608299.80 17347239 PMC1819511

[B5] Blanco E. Shen H. Ferrari M. (2015). Principles of nanoparticle design for overcoming biological barriers to drug delivery. *Nat. Biotechnol.* 33:941. 10.1038/nbt.3330 26348965 PMC4978509

[B6] Caraglia M. Luongo L. Salzano G. Zappavigna S. Marra M. Guida F. (2013). Stealth liposomes encapsulating zoledronic acid: a new opportunity to treat neuropathic pain. *Mol. Pharm.* 10 1111–1118. 10.1021/mp3006215 23327778

[B7] De Williams A. C. Eccleston C. Morley S. (2012). Psychological therapies for the management of chronic pain (excluding headache) in adults. *Cochr. Data. Syst. Rev.* 11:CD007407. 10.1002/14651858.CD007407.pub3 PMC6483325 23152245

[B8] Ducic I. Mesbahi A. N. Attinger C. E. Graw K. (2008). The role of peripheral nerve surgery in the treatment of chronic pain associated with amputation stumps. *Plastic Reconst. Surgery* 121 908–914. 10.1097/01.prs.0000299281.57480.77 18317139

[B9] Durán-Lobato M. Martín-Banderas L. Gonçalves L. M. Fernández-Arévalo M. Almeida A. J. (2015). Comparative study of chitosan-and PEG-coated lipid and PLGA nanoparticles as oral delivery systems for cannabinoids. *J. Nanop. Res.* 17:61.

[B10] Farokhzad O. C. Langer R. (2009). Impact of nanotechnology on drug delivery. *ACS Nano* 3 16–20. 19206243 10.1021/nn900002m

[B11] Feynman R. P. (1960). *There’s Plenty of Room at the Bottom.* Pasadena, CA: California Institute of Technology.

[B12] Francis R. Aspray T. Hide G. Sutcliffe A. Wilkinson P. (2008). Back pain in osteoporotic vertebral fractures. *Osteopor. Int.* 19 895–903. 10.1007/s00198-007-0530-x 18071648

[B13] Gao Y.-J. Ji R.-R. (2010). Targeting astrocyte signaling for chronic pain. *Neurotherapeutics* 7 482–493. 10.1016/j.nurt.2010.05.016 20880510 PMC2950097

[B14] Garcia X. Escribano E. Domenech J. Queralt J. Freixes J. (2011). In vitro characterization and in vivo analgesic and anti-allodynic activity of PLGA-bupivacaine nanoparticles. *J. Nanopar. Res.* 13 2213–2223. 10.1007/s11051-010-9979-1

[B15] Gdowski A. S. Ranjan A. Sarker M. R. Vishwanatha J. K. (2017). Bone-targeted cabazitaxel nanoparticles for metastatic prostate cancer skeletal lesions and pain. *Nanomedicine* 12 2083–2095. 10.2217/nnm-2017-0190 28805551 PMC5585843

[B16] Gu Y. H. Yan X. B. Huang D. Han R. Wu L. X. (2012). *NR2B-siRNA Mediated by Hydroxyapatite Nanoparticles Relieves for Malin-Induced Pain of Mice.* Pfaffikon: Advanced Materials Research, Trans Tech Publications.

[B17] Gwak Y. S. Hassler S. E. Hulsebosch C. E. (2013). Reactive oxygen species contribute to neuropathic pain and locomotor dysfunction via activation of CamKII in remote segments following spinal cord contusion injury in rats. *PAIN^®^* 154 1699–1708. 10.1016/j.pain.2013.05.018 23707296

[B18] Hoekman J. D. Srivastava P. Ho R. J. (2014). Aerosol-stable peptide-coated liposome nanoparticles: A proof-of-concept study with opioid fentanyl in enhancing analgesic effects and reducing plasma drug exposure. *J. Pharm. Sci.* 103 2231–2239. 10.1002/jps.24022 24909764 PMC4115018

[B19] Hua S. Cabot P. J. (2013). Targeted nanoparticles that mimic immune cells in pain control inducing analgesic and anti-inflammatory actions: a potential novel treatment of acute and chronic pain conditions. *Pain Physician* 16 E199–E216. 23703419

[B20] Ji R.-R. Xu Z.-Z. Gao Y.-J. (2014). Emerging targets in neuroinflammation-driven chronic pain. *Nat. Rev. Drug Dis.* 13 533–548. 10.1038/nrd4334 24948120 PMC4228377

[B21] Kaushik S. Hord A. H. Denson D. D. McAllister D. V. Smitra S. Allen M. G. (2001). Lack of pain associated with microfabricated microneedles. *Anesth. Anal.* 92 502–504. 10.1097/00000539-200102000-00041 11159258

[B22] Kaye A. D. Cornett E. M. Hart B. Patil S. Pham A. Spalitta M. (2018). Novel pharmacological nonopioid therapies in chronic pain. *Curr. Pain Head. Rep.* 22:31. 10.1007/s11916-018-0674-8 29616344

[B23] Koudelka Š Turánek J. (2012). Liposomal paclitaxel formulations. *J. Control. Release* 163 322–334. 10.1016/j.jconrel.2012.09.006 22989535

[B24] Lalani J. Patil S. Kolate A. Lalani R. Misra A. (2015). Protein-functionalized PLGA nanoparticles of lamotrigine for neuropathic pain management. *AAPS Pharm. Sci.Tech.* 16 413–427. 10.1208/s12249-014-0235-3 25354788 PMC4370975

[B25] Liu Q. Jin L. Mahon B. H. Chordia M. D. Shen F. H. Li X. (2013). A novel treatment of neuroinflammation against low back pain by soluble fullerol nanoparticles. *Spine* 38:1443. 10.1097/brs.0b013e31828fc6b7 23466506 PMC3731423

[B26] Mahmoudi M. Bertrand N. Zope H. Farokhzad O. C. (2016). Emerging understanding of the protein corona at the nano-bio interfaces. *Nano Today* 11 817–832. 10.1016/j.nantod.2016.10.005

[B27] Makadia H. K. Siegel S. J. (2011). Poly lactic-co-glycolic acid (PLGA) as biodegradable controlled drug delivery carrier. *Polymers* 3 1377–1397. 10.3390/polym3031377 22577513 PMC3347861

[B28] Malek N. Mrugala M. Makuch W. Kolosowska N. Przewlocka B. Binkowski M. (2015). A multi-target approach for pain treatment: dual inhibition of fatty acid amide hydrolase and TRPV1 in a rat model of osteoarthritis. *Pain* 156 890–903. 10.1097/j.pain.0000000000000132 25719612

[B29] Mantyh P. W. Clohisy D. R. Koltzenburg M. Hunt S. P. (2002). Molecular mechanisms of cancer pain. *Nat. Rev. Cancer* 2 201–209. 11990856 10.1038/nrc747

[B30] Nyström A. M. Fadeel B. (2012). Safety assessment of nanomaterials: implications for nanomedicine. *J. Control. Release* 161 403–408. 10.1016/j.jconrel.2012.01.027 22306428

[B31] Palazzolo S. Bayda S. Hadla M. Caligiuri I. Corona G. Toffoli G. (2018). The clinical translation of organic nanomaterials for cancer therapy: a focus on polymeric nanoparticles, micelles, liposomes and exosomes. *Curr. Med. Chem.* 25 4224–4268. 10.2174/0929867324666170830113755 28875844

[B32] Park J.-H. Gu L. Von Maltzahn G. Ruoslahti E. Bhatia S. N. Sailor M. J. (2009). Biodegradable luminescent porous silicon nanoparticles for in vivo applications. *Nat. Mater.* 8 331–336. 10.1038/nmat2398 19234444 PMC3058936

[B33] Patel D. Naik S. Misra A. (2012). Improved transnasal transport and brain uptake of tizanidine HCl-loaded thiolated chitosan nanoparticles for alleviation of pain. *J. Pharm. Sci.* 101 690–706. 10.1002/jps.22780 22006260

[B34] Pohl M. Smith L. (2012). Chronic pain and addiction: challenging co-occurring disorders. *J. Psych. Drugs* 44 119–124. 10.1080/02791072.2012.684621 22880539

[B35] Ramírez-García P. D. Retamal J. S. Shenoy P. Imlach W. Sykes M. Truong N. (2019). A pH-responsive nanoparticle targets the neurokinin 1 receptor in endosomes to prevent chronic pain. *Nat. Nanotechnol.* 14 1150–1159. 10.1038/s41565-019-0568-x 31686009 PMC7765343

[B36] Said G. (2007). Diabetic neuropathy—a review. *Nat. Clin. Prac. Neurol.* 3 331–340. 10.1038/ncpneuro0504 17549059

[B37] Shen H. Hu X. Szymusiak M. Wang Z. J. Liu Y. (2013). Orally administered nanocurcumin to attenuate morphine tolerance: comparison between negatively charged PLGA and partially and fully PEGylated nanoparticles. *Mol. Pharm.* 10 4546–4551. 10.1021/mp400358z 24195658 PMC3939066

[B38] Shin J. Yin Y. Park H. Park S. Triantafillu U. L. Kim Y. (2018). p38 siRNA-encapsulated PLGA nanoparticles alleviate neuropathic pain behavior in rats by inhibiting microglia activation. *Nanomedicine* 13 1607–1621. 10.2217/nnm-2018-0054 30028250

[B39] Smith L. J. Valenzuela J. R. Krugner-Higby L. A. Brown C. Heath T. D. (2006). A single dose of liposome-encapsulated hydromorphone provides extended analgesia in a rat model of neuropathic pain. *Compar. Med.* 56 487–492. 17219779

[B40] Song T. Gu K. Wang W. Wang H. Yang Y. Yang L. (2015). Prolonged suppression of neuropathic pain by sequential delivery of lidocaine and thalidomide drugs using PEGylated graphene oxide. *J. Pharm. Sci.* 104 3851–3860. 10.1002/jps.24598 26220057

[B41] Sullivan M. D. Howe C. Q. (2013). Opioid therapy for chronic pain in the United States: promises and perils. *PAIN^®^* 154 S94–S100. 24036286 10.1016/j.pain.2013.09.009 PMC4204477

[B42] Tang Z. Zhao P. Ni D. Liu Y. Zhang M. Wang H. (2018). Pyroelectric nanoplatform for NIR-II-triggered photothermal therapy with simultaneous pyroelectric dynamic therapy. *Mat. Horiz.* 5 946–952. 10.1039/c8mh00627j

[B43] Tosi G. Costantino L. Rivasi F. Ruozi B. Leo E. Vergoni A. V. (2007). Targeting the central nervous system: in vivo experiments with peptide-derivatized nanoparticles loaded with Loperamide and Rhodamine-123. *J. Control. Release* 122 1–9. 10.1016/j.jconrel.2007.05.022 17651855

[B44] Urie R. Ghosh D. Ridha I. Rege K. (2018). Inorganic nanomaterials for soft tissue repair and regeneration. *Ann. Rev. Biomed. Eng.* 20 353–374. 10.1146/annurev-bioeng-071516-044457 29621404

[B45] Van Dommelen S. M. Vader P. Lakhal S. Kooijmans S. van Solinge W. W. Wood M. J. (2012). Microvesicles and exosomes: opportunities for cell-derived membrane vesicles in drug delivery. *J. Control. Release* 161 635–644. 10.1016/j.jconrel.2011.11.021 22138068

[B46] Ventola C. L. (2017). Progress in nanomedicine: approved and investigational nanodrugs. *Pharmacy Therapeutics* 42:742. PMC5720487 29234213

[B47] Volkow N. Benveniste H. McLellan A. T. (2018). Use and misuse of opioids in chronic pain. *Ann. Rev. Med.* 69 451–465. 10.1146/annurev-med-011817-044739 29029586

[B48] Volkow N. D. McLellan A. T. (2016). Opioid abuse in chronic pain—misconceptions and mitigation strategies. *New Eng. J. Med.* 374 1253–1263. 10.1056/nejmra1507771 27028915

[B49] Vowles K. E. McEntee M. L. Julnes P. S. Frohe T. Ney J. P. van der Goes D. N. (2015). Rates of opioid misuse, abuse, and addiction in chronic pain: a systematic review and data synthesis. *Pain* 156 569–576. 10.1097/01.j.pain.0000460357.01998.f1 25785523

[B50] Wu P.-C. Hsiao H.-T. Lin Y.-C. Shieh D.-B. Liu Y.-C. (2017). The analgesia efficiency of ultrasmall magnetic iron oxide nanoparticles in mice chronic inflammatory pain model. *Nanomed. Nanotechnol. Biol. Med.* 13 1975–1981. 10.1016/j.nano.2017.05.005 28539274

[B51] Xu C. Lei C. Yu C. (2019). Mesoporous silica nanoparticles for protein protection and delivery. *Front. Chem.* 7:290. 10.3389/fchem.2019.00290 PMC6504683 31119124

[B52] Zhang C. Bu W. Ni D. Zhang S. Li Q. Yao Z. (2016). Synthesis of iron nanometallic glasses and their application in cancer therapy by a localized Fenton reaction. *Angew. Chem. Int. Ed.* 55 2101–2106. 10.1002/anie.201510031 26836344

[B53] Zhang J.-M. An J. (2007). Cytokines, inflammation and pain. *Int. Anesthesiol. Clin.* 45:27. 10.1097/aia.0b013e318034194e 17426506 PMC2785020

[B54] Zhuo M. (2016). Neural mechanisms underlying anxiety–chronic pain interactions. *Trends Neurosci.* 39 136–145. 10.1016/j.tins.2016.01.006 26878750

